# N-glycosylation of UNC93B1 at a Specific Asparagine Residue Is Required for TLR9 Signaling

**DOI:** 10.3389/fimmu.2022.875083

**Published:** 2022-07-07

**Authors:** Hyun-Sup Song, Soeun Park, Ji-Won Huh, Yu-Ran Lee, Da-Jung Jung, Chorong Yang, So Hyun Kim, Ho Min Kim, You-Me Kim

**Affiliations:** ^1^ Graduate School of Medical Science and Engineering, Korea Advanced Institute of Science and Technology (KAIST), Daejeon, South Korea; ^2^ Department of Systems Biology, College of Life Science and Biotechnology, Yonsei University, Seoul, South Korea; ^3^ Division of Integrative Biosciences and Bioengineering, Pohang University of Science and Technology (POSTECH), Pohang, South Korea; ^4^ Center for Biomolecular & Cellular Structure, Institute for Basic Science (IBS), Daejeon, South Korea

**Keywords:** toll-like receptor, UNC93B1, N-glycosylation, MyD88, endosome, nucleotide, TLR9, TLR7

## Abstract

Toll-like receptors (TLRs) play critical roles in the first line of host defense against pathogens through recognition of pathogen-associated molecular patterns and initiation of the innate immune responses. The proper localization of TLRs in specific subcellular compartments is crucial for their ligand recognition and downstream signaling to ensure appropriate responses against pathogens while avoiding erroneous or excessive activation. Several TLRs, including TLR7 and TLR9 but not TLR4, depend on UNC93B1 for their proper intracellular localization and signaling. Accumulating evidence suggest that UNC93B1 differentially regulates its various client TLRs, but the specific mechanisms by which UNC93B1 controls individual TLRs are not well understood. Protein N-glycosylation is one of the most frequent and important post-translational modification that occurs in membrane-localized or secreted proteins. UNC93B1 was previously shown to be glycosylated at Asn251 and Asn272 residues. In this study, we investigated whether N-glycosylation of UNC93B1 affects its function by comparing wild type and glycosylation-defective mutant UNC93B1 proteins. It was found that glycosylation of Asn251 and Asn272 residues can occur independently of each other and mutation of neither N251Q or N272Q in UNC93B1 altered expression and localization of UNC93B1 and TLR9. In contrast, CpG DNA-stimulated TLR9 signaling was severely inhibited in cells expressing UNC93B1(N272Q), but not in cells with UNC93B1(N251Q). Further, it was found that glycosylation at Asn272 of UNC93B1 is essential for the recruitment of MyD88 to TLR9 and the subsequent downstream signaling. On the other hand, the defective glycosylation at Asn272 did not affect TLR7 signaling. Collectively, these data demonstrate that the glycosylation at a specific asparagine residue of UNC93B1 is required for TLR9 signaling and the glycosylation status of UNC93B1 differently affects activation of TLR7 and TLR9.

## Introduction

Toll-like receptors (TLRs) play critical roles in the first line of host defense against pathogens through recognition of pathogen-associated molecular patterns and initiation of the innate immune responses (1, 2). The proper localization of TLRs in specific subcellular compartments is crucial for their ligand recognition and downstream signaling to ensure appropriate responses against pathogens (3, 4). Nucleotide-sensing TLRs, such as TLR 3, 7, 8, 9, and 13, are generally located in endosomes in which they are activated by DNA or RNA released from internalized virus or phagocytosed bacteria while avoiding unwanted activation by host-derived nucleic acids that are abundantly present in the extracellular space (3–5). Aberrant localization and activation of nucleotide-sensing TLRs have been associated with emergence of autoinflammatory or autoimmune diseases such as systemic lupus erythematosus and arthritis (6–9).

Intracellular trafficking and signaling of nucleotide-sensing TLRs are strictly dependent on the multipass membrane protein UNC93B1. UNC93B1 physically interacts with nucleotide-sensing TLRs to mediate their exit from the endoplasmic reticulum (ER) *via* COP II vesicles and the subsequent trafficking to endosomes (10–12). UNC93B1 not only facilitates the trafficking of nucleotide-sensing TLRs but also serves as a chaperone and stabilizes TLRs (13). A missense mutation causing the change of His412 to Arg (H412R) in UNC93B1 abolishes the interaction of UNC93B1 with all nucleotide-sensing TLRs, resulting in retention of the TLRs in the ER and the complete loss of their activation. Consequently, mice bearing the H412R mutation of UNC93B1 show compromised immune responses against various microbes (14). In humans, autosomal recessive deletion of UNC93B1 gene was found in children suffering from sporadic herpes simplex virus 1-associated encephalitis and autosomal dominant deficiency was recently detected in SARS-CoV-2-infected patients suffering from life-threatening pneumonia, demonstrating the physiological importance of UNC93B1-TLR interaction in host defense (14–16). In addition to regulating the nucleotide-sensing TLRs, UNC93B1 is also necessary for endosomal localization of TLR11 and TLR12, both recognizing the bacterial protein profilin, and for cell surface localization of TLR5 sensing the bacterial protein flagellin (17–19).

Despite the common involvement of UNC93B1 in regulating the subcellular localization and signaling of many TLRs, the manner by which UNC93B1 controls individual TLRs seem divergent in detail. The first evidence for the differential regulation of particular TLRs by UNC93B1 came from the study demonstrating that the point mutation of Asp34 to Ala (D34A) in the N-terminal tail of UNC93B1 biases TLR responses toward activation of TLR7 over TLR9 (20). Compared to wild type (WT) UNC93B1, the D34A mutant UNC93B1 protein binds better to TLR7 and less to TLR9 and facilitates the ER exit of TLR7 at the expense of TLR9, which results in TLR7- and type I interferon-dependent systemic inflammation in mice (20, 21). These findings suggest that binding of UNC93B1 to individual TLRs may be differentially regulated.

The tyrosine-based sorting motif, Tyr-x-x-Φ (YxxΦ, x is any amino residue and Φ represents bulky hydrophobic residue) in the C-terminal tail of UNC93B1 mediates the interaction with the μ subunit of the clathrin adaptor protein 2 (AP2) complex (12, 22). The mutation of Tyr539 to Ala (Y539A) in the YxxΦ motif of UNC93B1 inhibits the endosomal localization of TLR9 without affecting the localization of other UNC93B1-interacting TLRs in murine macrophages, demonstrating a unique sorting mechanism employed by UNC93B1 for TLR9 (12). Unlike in murine cells, mutation of the tyrosine-based sorting motif (YxxΦ to AxxA) of human UNC93B1 resulted in accumulation of UNC93B1 at the cell surface and inhibition of signaling by TLR7, 8, and 9 (22). Once in endosomes, UNC93B1 releases TLR9 and the separation of TLR9 from UNC93B1 was suggested to be critical for the DNA binding and signaling of the receptor (23). Similar to TLR9, TLR3 is also released from UNC93B1 in endosomes (23). In contrast, TLR7 seems to remain bound to UNC93B1 and UNC93B1 recruits syntenin-1 *via* its C-terminal tail, which facilitates sorting of TLR7 into intraluminal vesicles of multivesicular bodies and terminates its signaling (24). Binding of syntenin-1 is regulated by phosphorylation of two serine residues in the C-tail of UNC93B1 and inhibition of the UNC93B1/syntenin-1 interaction or K63-ubiquitination of UNC93B1 results in enhancement of TLR7 signaling and causes autoimmunity in mice (24). Collectively, these studies demonstrate that UNC93B1 undergoes several post-translational modifications and utilizes various mechanisms to selectively control its client TLRs.

Protein N-glycosylation is one of the most frequent and important post-translational modification that occurs in membrane-localized or secreted proteins. It alters various biological properties of proteins: folding, stability, trafficking, subcellular localization, interaction with other proteins, and functions such as signal transduction (25–28). The first step of protein N-glycosylation is the co-translational attachment of a preformed ‘precursor oligosaccharide’ (composed of a total of 14 sugars including N-acetylglucosamine, mannose, and glucose) to an asparagine residue in the conserved N-glycosylation motif of Asn-x-Ser/Thr (NxS/T, x is any amino acid residue except for Pro) in the rough ER lumen. The oligosaccharide is further processed in the ER and the Golgi apparatus by trimming and addition of different sugars in highly ordered steps to finally form a ‘complex oligosaccharide’.

UNC93B1 was previously shown to be glycosylated at Asn251 and Asn272 residues, but the roles of N-glycosylation in regulating stability and function of UNC93B1 are unknown (12, 29). In this study, we analyzed whether N-glycosylation of UNC93B1 affects expression and localization of UNC93B1 and its client TLRs by comparing wild type and glycosylation-defective mutant UNC93B1 proteins. While no gross abnormalities of the mutant UNC93B1 proteins were found in terms of their expression, TLR interaction, and trafficking, defective glycosylation at Asn272, but not at Asn251, of UNC93B1 resulted in inhibition of TLR9 signaling, suggesting that N-glycosylation at a specific asparagine residue is essential for proper TLR9 signaling. Notably, TLR7 signaling was not affected by the defective glycosylation of UNC93B1 at Asn272. These results present yet another example how UNC93B1 differentially regulates signaling of TLR7 and TLR9.

## Results

**Figure 1:**
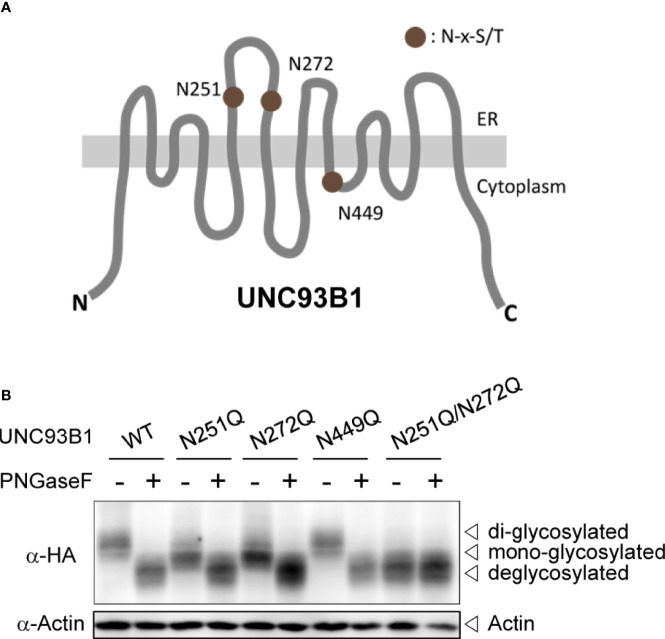
UNC93B1 is glycosylated at N251 and N272 residues. **(A)** Schematic diagram of UNC93B1. N-glycosylation sites predicted by the consensus amino acid sequence motif, Asn-x-Ser/Thr (NxS/T) are depicted. **(B)** HEK293T cell lysates expressing wild type or mutant UNC93B1-HA were treated with or without PNGase F. UNC93B1-HA and actin were detected by immunoblotting with anti-HA antibody and anti-actin antibody, respectively.

The full-length UNC93B1 is composed of 12 transmembrane helices (TM) and has three putative N-glycosylation sites conforming to the canonical N-x-S/T motif (**Figure 1A**). Among the three Asn residues, Asn251 and Asn272 are found in the loop between TM5 and TM6 and shown to be glycosylated in a cryo-EM structure study of UNC93B1 (29). Another residue, Asn449 is facing the cytoplasm and therefore not glycosylated. In order to investigate the functional role of N-glycosylation in UNC93B1, we individually mutated the three Asn residues to Gln (N251Q, N272Q, and N449Q) in the full length UNC93B1-HA and UNC93B1-GFP fusion proteins. We also generated UNC93B1 with a double mutation, N251Q/N272Q.

We first analyzed expression of each UNC93B1 mutant protein by immunoblotting (**Figure 1B**). Both UNC93B1(N251Q) and UNC93B1(N272Q) mutants showed a slight mobility shift compared to UNC93B1(WT), suggesting that the mutants have a lower molecular weight than the WT protein likely due to a partial lack of glycosylation. UNC93B1(N251Q/N272Q) showed a greater mobility shift. The mobility differences of WT and mutant UNC93B1 proteins disappeared when they were treated with Protein-N-glycosidase F (PNGase F), confirming that the mobility differences are due to different levels of glycosylation. Moreover, the mobility of UNC93B1(N251Q/N272Q) did not change after the PNGase F treatment, demonstrating that N251 and N272 are the only residues that are glycosylated in UNC93B1. These results also show that glycosylation status at N251 does not affect glycosylation at N272, and vice versa. Therefore, glycosylation of N251 and N272 can happen independently of each other.

**Figure 2:**
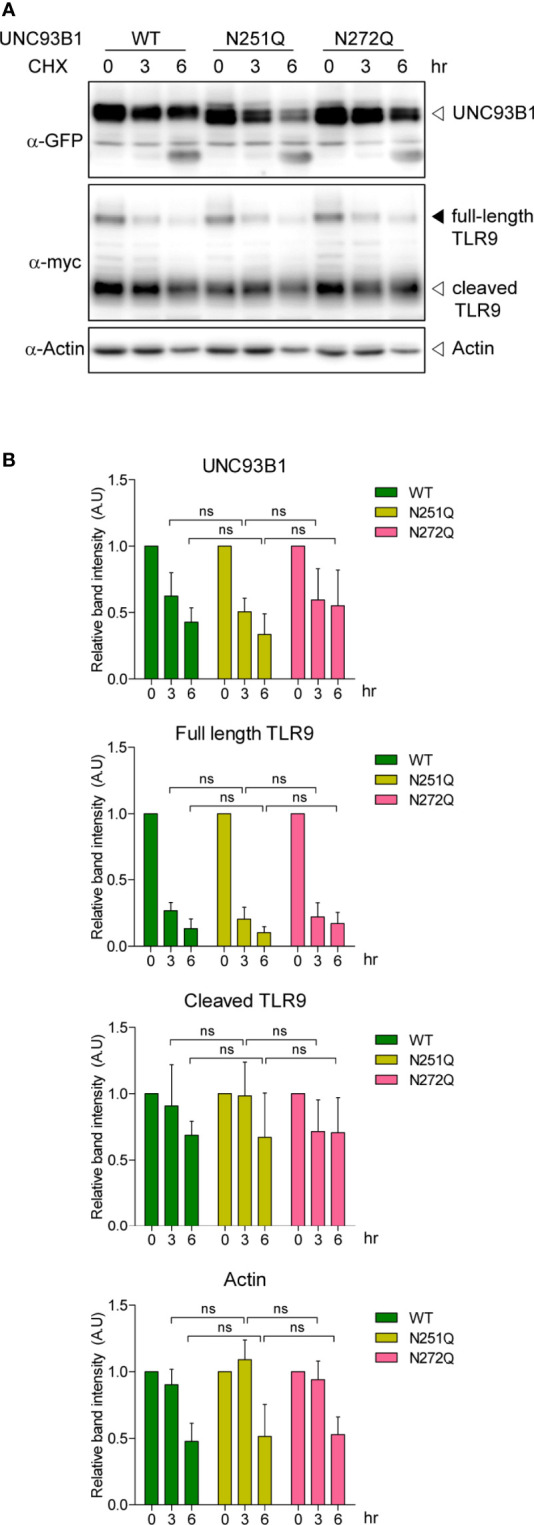
N-glycosylation of UNC93B1 does not affect the stability of UNC93B1 and TLR9. **(A)** Wild type or mutant UNC93B1-GFP were expressed in UNC93B1 KO iBMDMs together with TLR9-myc. After inhibition of protein synthesis with 20 μg/ml cycloheximide for indicated time periods, cells were lysed and the lysates were loaded on an SDS-PAGE gel. After electrophoresis and protein transfer, UNC93B1-GFP, TLR9-myc, and actin in a single transfer membrane were sequentially detected by immunoblotting with anti-GFP, anti-myc, and anti-actin antibody, respectively. **(B)** The relative band intensity of UNC93B1, full-length TLR9, cleaved TLR9, and actin at each time point was calculated by dividing the band intensity of individual protein at the indicated time point with that at 0 h. Data represent means ± SD (n = 3). ns, not significant (Student's *t*-test).

N-glycosylation often plays important roles in proper protein folding and quality control of target proteins in the ER (25–28). It can also regulate protein stability and defective glycosylation at even a single Asn residue among many glycosylation sites can result in rapid protein degradation (26, 30, 31). To test if the glycosylation status affects stability of UNC93B1, WT or mutant UNC93B1-GFP together with TLR9-myc were expressed in immortalized bone marrow-derived macrophages (iBMDM) that were generated from UNC93B1-deficient (UNC93B1 KO) mice (13). Cells were then treated with cycloheximide (CHX) for various time periods to block new protein synthesis and protein level changes of UNC93B1 and TLR9 were assessed by immunoblotting. The level of UNC93B1(WT) decreased in a time-dependent manner after the CHX treatment. Neither UNC93B1(N251Q) nor UNC93B1(N272Q) mutant showed a statistically significant change in the decay pattern compared to UNC93B1(WT), suggesting that the partial lack of glycosylation does not impact the stability of UNC93B1 (**Figure 2**). Expression of TLR9 which requires functional UNC93B1 for its stability and trafficking to endosomes was also assessed (11, 13). At steady state, TLR9 exists as a full-length form and a cleaved form. The full-length TLR9 synthesized in the ER moves to endosomes where acidic proteases cleave the Z loop in the leucine-rich repeat domain of TLR9 to generate N-terminal and C-terminal fragments of TLR9 (32–34). This cleavage event is essential for efficient ligand recognition and activation of the receptor and also reflects the UNC93B1-assisted endosomal localization of TLR9 (32–34). The full-length TLR9 in cells expressing UNC93B1(WT) rapidly decreased after CHX treatment due to blockade of new protein synthesis and trafficking of the pre-existing TLR9 to endosomes which leads to its subsequent proteolytic processing (**Figure 2**). Because of the greater excess of cleaved TLR9 over the full-length form at steady state, an increase in the level of cleaved TLR9 after CHX treatment was not evident and rather it slightly decreased, mostly likely due to endosomal degradation of cleaved TLR9. The disappearance rate of the full-length and the cleaved TLR9 in cells expressing UNC93B1(N251Q) or UNC93B1(N272Q) was comparable to that in cells expressing UNC93B1(WT), implying that the defective glycosylation at each Asn residue of UNC93B1 does not significantly alter stability, trafficking, and processing of TLR9.

**Figure 3:**
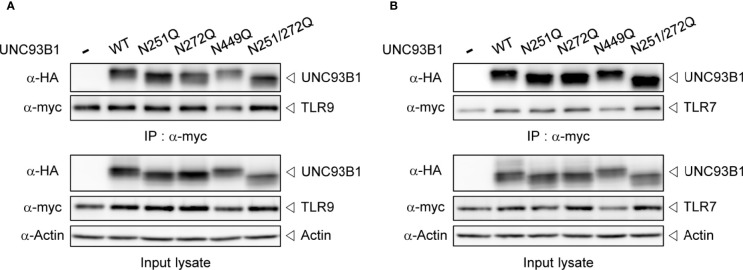
Interaction between UNC93B1 and nucleotide-sensing TLRs is not affected by N-glycosylation of UNC93B1. Wild type or mutant UNC93B1-HA were expressed in HEK293T cells together with TLR9-myc **(A)** or TLR7-myc **(B)**. After immunoprecipitation of TLRs with anti-myc antibody, TLRs and TLR-associated UNC93B1 in the immunoprecipitates was detected with anti-myc and anti-HA antibody, respectively (upper two panels). Expression levels of UNC93B1, TLRs, and actin in the cell lysates were probed with anti-HA, anti-myc, and anti-actin antibody, respectively (lower three panels).

Next, it was examined whether the physical interaction between UNC93B1 and TLR9 is affected by glycosylation status of UNC93B1 using a co-immunoprecipitation assay. WT or mutant UNC93B1-HA was expressed with TLR9-myc and TLR9-myc was immunoprecipitated with anti-myc antibody. The amount of UNC93B1 recovered in the immunoprecipitates of TLR9 were comparable regardless of the glycosylation site mutations, suggesting that the lack of glycosylation does not affect the interaction between UNC93B1 and TLR9 (**Figure 3A**). Similarly, mutation of glycosylation sites in UNC93B1 did not inhibit its interaction with TLR7 (**Figure 3B**).

**Figure 4:**
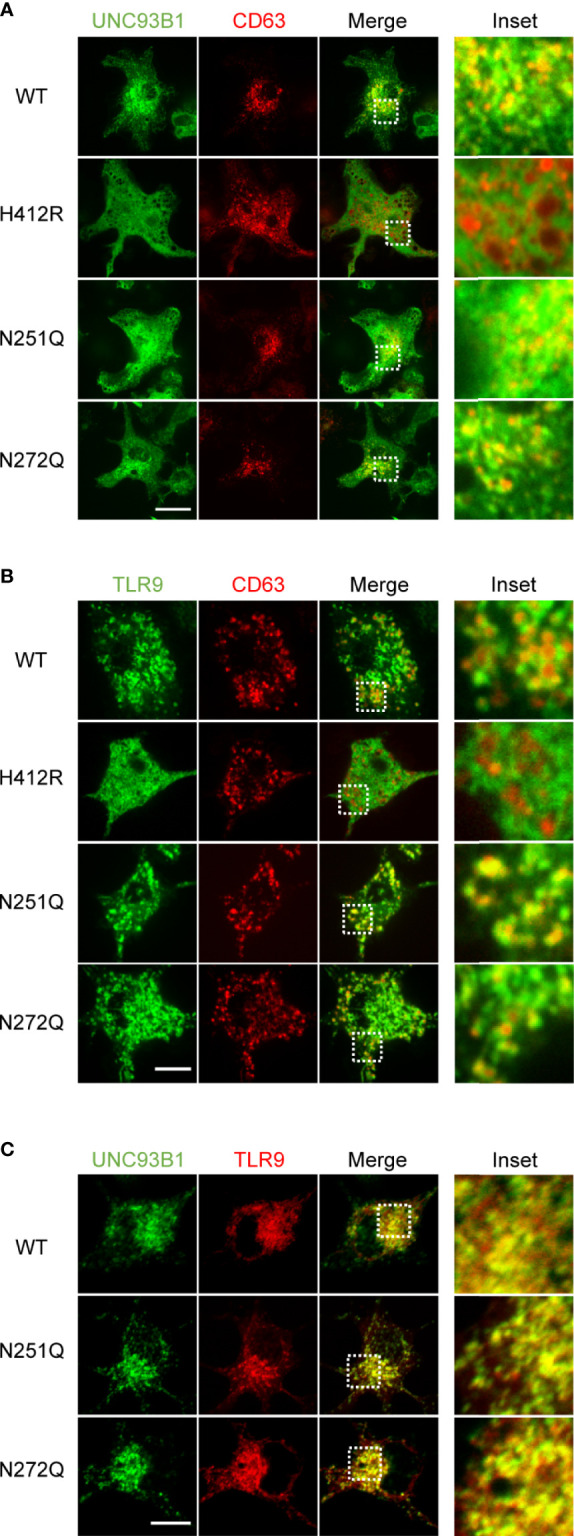
Intracellular localization of UNC93B1 and TLR9 is unaffected by defective N-glycosylation of UNC93B1. **(A)** UNC93B1-GFP (wild type or mutant) and CD63-cherry were expressed in UNC93B1 KO BMDCs and cells were imaged by confocal microscopy. Scale bar, 10 μm **(B)** TLR9-GFP and CD63-cherry were expressed in UNC93B1 KO BMDCs together with either wild type or mutant UNC93B1-HA. Localization of TLR9-GFP and CD63-cherry were detected by confocal microscopy. Scale bar, 5 μm **(C)** Wild type or mutant UNC93B1-GFP were expressed in UNC93B1 KO BMDCs together with TLR9-myc. After fixation and permeabilization, cells were stained with anti-myc antibody and Alexa Fluor 647-conjugated secondary antibody and imaged by confocal microscopy. Scale bar, 10 μm.

Further, intracellular localization of UNC93B1(N251Q) and UNC93B1(N272Q) was analyzed by confocal microscopy. Similar to UNC93B1(WT), the two mutant UNC93B1 proteins were distributed in the ER network as well as in endosomes, suggesting that the glycosylation status of UNC93B1 does not affect its intracellular localization and trafficking (**Figure 4A**). The endosomal localization of UNC93B1 was shown by colocalization with the endosomal marker protein CD63. As previously shown, UNC93B1(H412R) was only found in the ER and did not colocalize with CD63 (11). Because there was no defect in the interaction of UNC93B1(N251Q) and UNC93B1(N272Q) with TLRs, the normal localization of TLR9 in cells expressing the mutant UNC93B1 proteins was anticipated. Indeed, TLR9 was properly localized in the CD63-positive endosomes in cells expressing either UNC93B1(N251Q) or UNC93B1(N272Q) as well as in cells expressing UNC93B1(WT) (**Figure 4B**). In contrast, TLR9 was not colocalized with CD63 in cells expressing UNC93B1(H412R) (11). These data are in accord with the earlier data showing that proteolytic processing of TLR9 occurs normally regardless of UNC93B1 glycosylation (**Figure 2**). As expected from normal interaction with TLR9, both UNC93B1(N251Q) and UNC93B1(N272Q) were extensively colocalized with TLR9 as UNC93B1(WT) did (**Figure 4C**).

**Figure 5:**
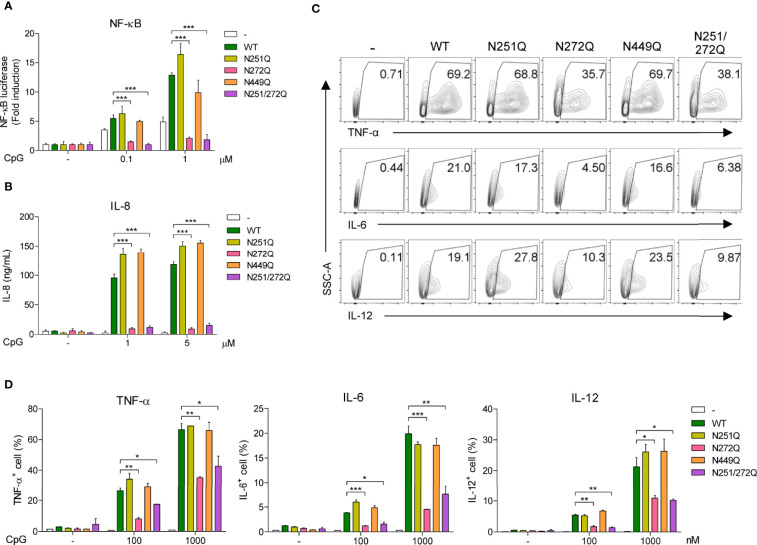
Defective glycosylation at N272 residue of UNC93B1 inhibits TLR9 signaling. **(A)** HEK293T cells expressing the NF-κB-luciferase reporter, TLR9-myc, and UNC93B1-HA (wild type or mutant) were stimulated with indicated concentration of CpG DNA for 6 h. After cell lysis, the luciferase activity was measured and the fold induction of luciferase in stimulated cells over unstimulated cells were depicted. **(B)** HEK293T cells expressing TLR9-myc and UNC93B1-HA (wild type or mutant) were stimulated with indicated concentration of CpG DNA for 24 h. IL-8 secreted in the cell supernatant was measured by ELISA. **(C, D)** GFP or UNC93B1-GFP (wild type or mutant) was expressed in UNC93B1 KO BMDCs. After stimulation of cells with CpG DNA for 4 h, cytokine production was analyzed by intracellular cytokine staining followed by flow cytometry. Representative FACS plots for TNF-α, IL-6, and IL-12 in GFP^+^ cells are shown **(C)**. The proportion of TNF-α ^+^, IL-6^+^, and IL-12^+^ cells among GFP^+^ populations are shown **(D)**. Data represent means ± SD (n = 3). *p < 0.05, **p < 0.01, ***p < 0.001 (Student’s *t*-test).

So far, these data suggest that defective glycosylation of UNC93B1 may not have any functional consequences. Nevertheless, we decided to analyze activation of TLR9 in cells expressing the mutant UNC93B1 proteins. First, TLR9 was expressed together with WT or mutant UNC93B1 proteins in HEK293T cells having an NF-κB-luciferase reporter. TLR9-mediated NF-κB activation was analyzed after cells were stimulated with CpG DNA (**Figure 5A**). Surprisingly, cells expressing either UNC93B1(N272Q) or UNC93B1(N251Q/N272Q) showed a severely blunted NF-κB activation upon CpG DNA stimulation whereas NF-κB activation in cells expressing UNC93B1(N251Q) or UNC93B1(N449Q) was normal as in cells expressing UNC93B1(WT). Similarly, CpG DNA-stimulated IL-8 secretion was abrogated in cells expressing UNC93B1(N272Q) or UNC93B1(N251Q/N272Q) (**Figure 5B**). These data suggest that glycosylation of N272, but not of N251, in UNC93B1 is essential for TLR9 signaling. The importance of glycosylation at N272 was confirmed by measuring cytokine production in primary mouse bone marrow-derived dendritic cells (BMDC). WT or mutant UNC93B1 proteins were reconstituted in UNC93B1 KO BMDC and cytokine production upon CpG DNA stimulation was measured by intracellular cytokine staining. Production of TNF-α, IL-6, and IL-12 were all significantly inhibited in cells expressing either UNC93B1(N272Q) or UNC93B1(N251Q/N272Q), whereas the cytokine responses were normal in cells expressing UNC93B1(N251Q) (**Figures 5C, D**; **Supplementary Figure 1C**). These data confirm that proper TLR9 signaling requires N-glycosylation of a specific residue, that is N272, of UNC93B1. In comparison, cytokine production upon TLR7 stimulation with R848 was normal in cells expressing UNC93B1(N272Q) or UNC93B1(N251Q/N272Q), suggesting that the requirement of glycosylation at N272 in UNC93B1 specifically applies to TLR9 signaling and not to TLR7 signaling (**Supplementary Figures 1A–C**). Intriguingly, it was repeatedly observed that TLR7-mediated cytokine production was upregulated in cells expressing UNC93B1(N251Q) (**Supplementary Figures 1A–C**). However, the effect of the N251Q mutation was not seen in the context of the N251Q/N272Q double mutation and we did not further investigate the role of N251 glycosylation of UNC93B1 on TLR7 signaling. As expected, neither N251Q or N272Q mutation affected LPS-stimulated, TLR4-mediated cytokine production (**Supplementary Figure 1C**).

**Figure 6:**
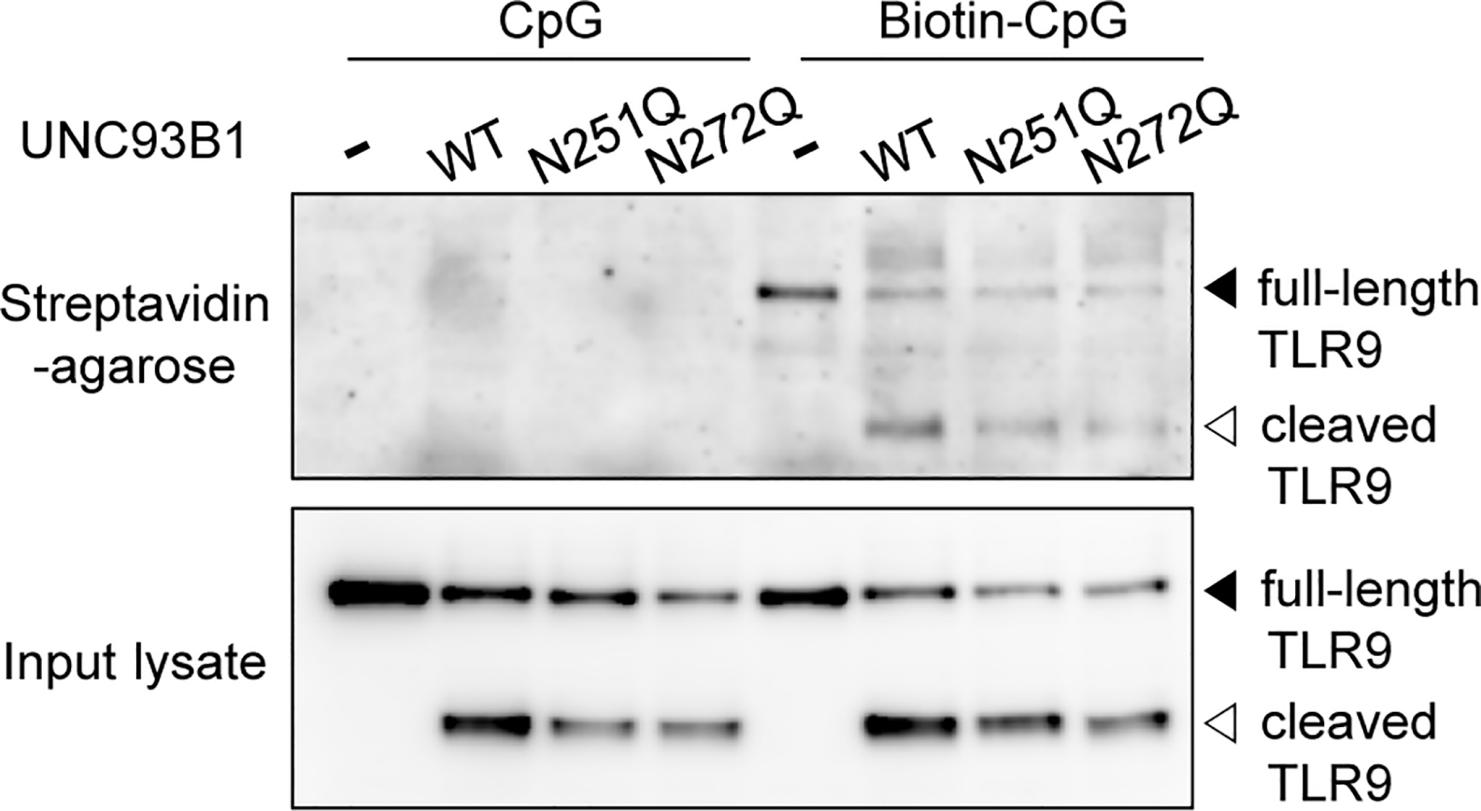
Defective N-glycosylation of UNC93B1 does not alter binding of TLR9 to CpG DNA. UNC93B1-GFP (wild type or mutant) and TLR9-myc were expressed in UNC93B1 KO iBMDMs. After stimulation with 1 μM CpG DNA or biotin-CpG DNA for 4 h, cells were lysed and biotin-CpG DNA was isolated with streptavidin-agarose beads. TLR9-myc bound to biotin-CpG DNA (top panel) or in the input cell lysates (bottom panel) was detected by immunoblotting with anti-myc antibody.

In order to gain more insight in how the selective defect of glycosylation at N272 inhibits TLR9 signaling, the interaction between TLR9 and its ligand was analyzed in cells expressing WT or mutant UNC93B1. Cells were treated with biotinylated-CpG DNA and the interaction between TLR9 and CpG DNA was assessed by immunoblotting of TLR9 after the CpG DNA pull-down with streptavidin-conjugated agarose beads (**Figure 6**). TLR9 was found to bind CpG DNA in cells expressing UNC93B1(N272Q) as well as in cells expressing UNC93B1(N251Q), suggesting that the UNC93B1(N272Q)-specific defect of TLR9 signaling is not caused by failure of ligand binding. No TLR9 was detected in samples treated with unbiotinylated CpG DNA, confirming the specificity of the assay.

**Figure 7:**
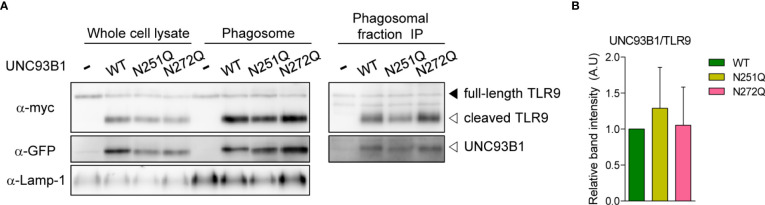
Association between UNC93B1 and TLR9 in phagosomes is unaffected by N-glycosylation of UNC93B1. **(A)** Phagosomes were isolated from UNC93B1 KO iBMDMs expressing TLR9-myc and UNC93B1-GFP (wild type or mutant). TLR9, UNC93B1, and Lamp-1 in whole cell lysates and isolated phagosomes were detected by immunoblotting with anti-myc, anti-GFP, and anti-LAMP-1 antibody, respectively (left panels). TLR9 was immunoprecipitated with anti-myc antibody from the isolated phagosomes and TLR9 and UNC93B1 in the immunoprecipitates were probed by anti-myc and anti-GFP antibody, respectively (right panels). **(B)** The ratio of TLR9-associated UNC93B1 was calculated by dividing the band intensity of UNC93B1-GFP with that of TLR9-myc in the immunoprecipitates of phagosomes. The relative levels of the UNC93B1/TLR9 ratio among samples were presented compared to that of UNC93B1(WT)/TLR9. Data are means ± SD of two independent experiments.

A recent study demonstrated that UNC93B1 is released from TLR9 once the TLR9-UNC93B1 complex reaches endosomes and phagosomes and the separation from UNC93B1 is required for proper signaling of TLR9 (23). Therefore, we analyzed whether UNC93B1(N272Q) has a defect in releasing TLR9 and shows a stronger interaction with TLR9 in phagosomes. For this, phagosomes were isolated from UNC93B1 KO cells expressing either wild type and mutant UNC93B1. Then, association between UNC93B1 and TLR9 was assessed by immunoprecipitation of TLR9 followed by immunoblotting of UNC93B1 (**Figure 7**). The majority of TLR9 was present as a cleaved form in phagosomes and TLR9 levels in phagosomes were equivalent regardless of the UNC93B1 mutations (**Figure 7A**, left panel). This indicates that the N272Q mutation of UNC93B1 does not cause a defect in TLR9 trafficking to phagosomes, as expected from normal TLR9 trafficking to endosomes in cells expressing UNC93B1(N272Q) (**Figure 4B**). On the other hand, the cleaved TLR9 was not detected in cells without UNC93B1 expression. Moreover, recovery of UNC93B1 in immunoprecipitates of TLR9 was comparable regardless of UNC93B1 mutations, demonstrating that the TLR9-UNC93B1 interaction in phagosomes is not affected by UNC93B1 glycosylation (**Figure 7A**, right panel and **7B**).

**Figure 8:**
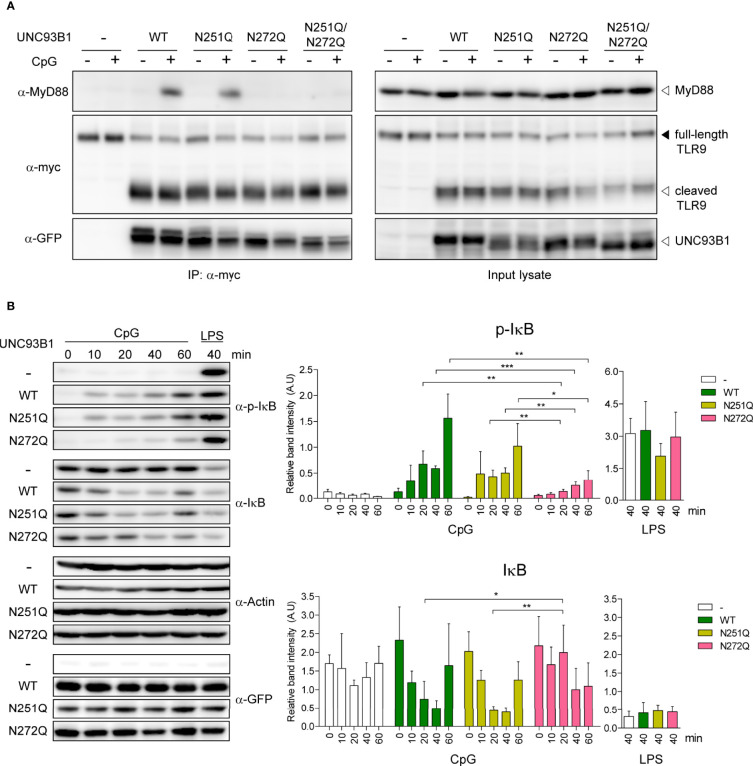
N-glycosylation of UNC93B1 at N272 is required for MyD88 recruitment and downstream signaling of TLR9. **(A)** UNC93B1-GFP (wild type or mutant) and TLR9-myc were expressed in UNC93B1 KO iBMDMs. After stimulation with 1 μM CpG DNA for 2 h, cells were lysed and TLR9-myc was immunoprecipitated from the cell lysates. TLR9, UNC93B1, and MyD88 were detected from the immunoprecipitates (left panels) and the input lysates (right panel) with anti-myc, anti-GFP, and anti-MyD88 antibody, respectively. **(B)** UNC93B1 KO iBMDMs expressing UNC93B1-GFP (wild type or mutant) and TLR9-myc were stimulated with 1 μM CpG DNA or 100 ng/ml LPS for indicated time periods. After cell lysis, phosphorylated IκB, total IκB, actin, and UNC93B-GFP were detected by immunoblotting with anti-phospho-IκB, anti-IκB, anti-actin, and anti-GFP antibody, respectively. Representative immunoblot images from four independent experiments were shown (left panels). Relative band intensity of phosphorylated IκB (upper right panel) and IκB (lower right panel) was calculated by dividing the band intensity of phosphorylated IκB and IκB with that of actin, respectively. Data are means ± SD (n = 4). *P < 0.05, ** < 0.01, ***P < 0.001 (Student's t-test).

Despite the significant impairment of TLR9-mediated NF-κB activation and cytokine responses in cells expressing UNC93B1(N272Q), stability and subcellular localization of both TLR9 and UNC93B1(N272Q) were not affected. Neither were the interactions between the two proteins and the ligand binding by TLR9. Therefore, we turned our attention to cytosolic signaling events induced by TLR9 stimulation. MyD88 is the key cytosolic adaptor molecule that is recruited to the Toll/interleukin-1 receptor (TIR) domain of most TLRs upon ligand binding to initiate intracellular signaling cascades (1). Therefore, TLR9-mediated recruitment of MyD88 was compared in cells expressing either WT or mutant UNC93B1 using a co-immunoprecipitation assay. In unstimulated conditions, no association between TLR9 and MyD88 was observed, whereas stimulation with CpG DNA resulted in robust association of TLR9 and MyD88 in cells expressing UNC93B1(WT) and UNC93B1(N251Q) (**Figure 8A**). In contrast, the recruitment of MyD88 to TLR9 was barely noticeable in cells expressing UNC93B1(N272Q) and UNC93B1(N251Q/N272Q), demonstrating that glycosylation at N272 of UNC93B1 is required for the recruitment of MyD88 by TLR9. We also analyzed phosphorylation and degradation of inhibitor of κB (Iκβ), the events that lead to freeing of NF-κB from Iκβ and its subsequent nuclear translocation for transcriptional activation of target genes such as inflammatory cytokines. CpG DNA-stimulated phosphorylation and subsequent degradation of Iκβ were significantly inhibited in cells expressing UNC93B1(N272Q) compared to cells with UNC93B1(WT) or UNC93B1(N251Q) (**Figure 8B**). As expected, LPS-stimulated phosphorylation and degradation of Iκβ was not influenced by UNC93B1 glycosylation. These data demonstrate that the specific glycosylation at N272 of UNC93B1 is crucial for recruitment of MyD88 to TLR9 and activation of the downstream signaling pathway.

## Discussion

Protein N-glycosylation is by far the most frequent and ubiquitous post-translational modification and it can significantly affect characteristics and functions of target proteins through addition of bulky, hydrophilic carbohydrate structures to specific asparagine residues. N-glycosylation was previously shown to regulate cell surface expression and signaling of TLRs (35, 36). In this study, we analyzed a role of N-glycosylation in UNC93B1 function by mutating N251 and N272 residues, the only two residues subjected to N-glycosylation in UNC93B1.

Against our initial expectation, we did not observe any major defects in stability, intracellular localization, and interaction with TLRs by mutation at either N251 or N272 residue of UNC93B1. In addition, stability and endosomal/phagosomal localization of TLR9 seem unaltered by the glycosylation site mutations of UNC93B1. Despite no apparent effects of faulty UNC93B1 glycosylation on both UNC93B1 and TLR9, we found that the N272Q mutation of UNC93B1 significantly inhibited TLR9 signaling measured by NK-κB activation and cytokine production after stimulation with CpG DNA. The N272Q mutation did not inhibit TLR7 signaling, demonstrating that the glycosylation of UNC93B1 at N272 is specifically required for TLR9 signaling. Mechanistically, we found that the deficient glycosylation at N272 blocks recruitment of MyD88 to TLR9 upon CpG DNA stimulation.

It is puzzling how the N-glycosylation on the luminal side of UNC93B1 manipulates binding between TLR9 and MyD88 which takes place in the cytoplasmic space. Several biochemical and structural studies have demonstrated that agonist binding to ectodomains of TLR dimers induces conformational changes that bring the C-terminal ends of the two TLR ectodomains close together (34, 37–39). The C-terminal end of the TLR ectodomain is connected to the TM domain *via* a short juxtamembrane loop and close positioning of the two TM domains of TLR dimers bring their cytosolic TIR domains together for MyD88 recruitment. We propose that glycosylation of N272 in UNC93B1 somehow regulates the CpG DNA-induced conformational changes of TLR9 and modulates MyD88 recruitment. The recent cryo-EM structure of UNC93B1 bound to TLR3 shows that sugars on N272 of UNC93B1 are located near a loop region (residues 679-683) of the leucine-rich repeat C-terminal (LRRCT) domain of TLR3 whereas sugars on N251 was facing away the TLR3 ectodomain (29). The cryo-EM structure of the UNC93B1-TLR7 complex in the same study shows that the ectodomain of TLR7 is tilted more toward the N272 of UNC93B1 compared to that of TLR3 (29). The UNC93B1-TLR9 structure is unavailable and, when we modeled the UNC93B1-TLR9 complex by overlaying the LRRCT domain (residues 769-805) of the TLR9 ectodomain structure to the TLR7 LRRCT domain (residues 787-828) in the UNC93B1-TLR7 complex, we found that the TLR9 ectodomain is tilted toward N272 of UNC93B1 even more than the TLR7 ectodomain, possibly explaining the specific effect of N272 glycosylation on TLR9 signaling (**Supplementary Figure 2**). Although no contact was observed between sugars attached to N272 of UNC93B1 with the TLR3 or TLR7 ectodomain, only a few core sugar residues, but not the whole complex oligosaccharide structures attached to UNC93B1, are revealed in the study (29). Therefore, we suppose that there is a possibility for the large complex oligosaccharide bound to N272 of UNC93B1 to make a direct interaction with the TLR9 ectodomain and regulate the conformational changes that orientate the TLR9 TIR domain for efficient MyD88 recruitment upon CpG DNA stimulation. However, this hypothesis is in disagreement with a previous study showing that UNC93B1 dissociates from TLR9 in endosomes for optimal CpG DNA binding and the subsequent downstream signaling (23). The ligand binding-induced dissociation of UNC93B1 from TLRs was also supported by the cryo-EM studies of the UNC93B1-TLR complexes (29). Therefore, we cannot rule out another possibility that the bulky carbohydrate structure attached to N272 exerts steric hindrance to facilitate the dissociation of TLR9 from UNC93B1 and promotes TLR9 signaling although the glycosylation status of UNC93B1 did not seem to affect the dissociation of the phagosomal UNC93B1-TLR9 complex in our assays. The exact molecular mechanism by which the glycosylation of N272 in UNC93B1 modulates the MyD88 recruitment to TLR9 needs to be elucidated by further studies. The structural information on the CpG DNA-bound TLR9 dimers including the TM and TIR domains will certainly help answering the question.

While defective glycosylation at N251 of UNC93B1 had no effect on TLR9 signaling, we noted that TLR7-mediated cytokine production was elevated in cells expressing UNC93B1(N251Q), but not in UNC93B1(N272Q) or UNC93B1(N251Q/N272Q), compared to cells expressing UNC93B1(WT). This effect was more pronounced especially when cells were stimulated with a lower dose of R848 (**Supplementary Figure 1**). Because the inhibitory effect of the N251Q mutation on TLR7 signaling was not seen in the context of the N251Q/N272Q double mutation, we did not further follow up the observation. However, we cannot dismiss a possible role of N251 glycosylation on TLR7 signaling. Another limitation of our study is that we mostly relied on qualitative assessment for determining the effect of UNC93B1 glycosylation on protein stability and interaction between UNC93B1 and TLR9 using immunoblotting and co-immunoprecipitation assays. More accurate, quantitative measurements using radioisotope-based metabolic labeling coupled with pulse-chase experiments and biophysical approaches may reveal subtle differences in stability and interaction of UNC93B1 and TLRs in cells expressing glycosylation-defective UNC93B1.

In conclusion, our present study demonstrates that the specific N-glycosylation at N272 of UNC93B1 is required for signaling of TLR9, but not for signaling of TLR7. To our knowledge, this is the first report showing the importance of glycosylation in UNC93B1’s function. Our results also reveal another mechanism by which UNC93B1 can differentially regulate TLR7 and TLR9 signaling.

## Materials and Methods

### Mice and Cell Lines

Wild type C57BL/6 mice were purchased from DBL (Korea) and UNC93B1-deficient (UNC93B1 KO) mice were previously described (19). Mice were housed in specific pathogen free (SPF) facility of Korea Institute of Advanced Science and Technology (KAIST) and Pohang University of Science and Technology (POSTECH). All mouse experiments were approved by the Institutional Animal Care and Use Committee of KAIST and POSTECH. UNC93B1 KO immortalized bone marrow-derived macrophages (iBMDMs) were a kind gift from Eicke Latz (University of Bonn, Germany) (13) and maintained in DMEM media supplemented with 10% fetal bovine serum (Welgene, Korea) and 1% penicillin/streptomycin (Welgene, Korea). Human embryonic kidney (HEK) 293T cells were maintained in DMEM media supplemented with 5% fetal bovine serum (Welgene, Korea). Cells stably expressing UNC93B1 and TLRs were generated by retroviral transduction and antibiotics selection as described below.

### Reagents and Antibodies

CpG oligodeoxynucleotide-1826 (CpG DNA) and biotin-CpG DNA were purchased from TIB Molbiol (Germany). R848 and LPS (*Escherichia coli* O111:B4) were from Enzo Life Sciences (USA) and Sigma-Aldrich (USA), respectively. Rat monoclonal anti-HA (3F10) was purchased from Roche (USA). Mouse monoclonal anti-GFP (B-2) was purchased from Santa Cruz (USA). Mouse monoclonal anti-myc (9B11), rabbit monoclonal anti-MyD88 (D80F5), mouse monoclonal anti-phospho-Iκβ (5A5), and anti-Iκβ were obtained from Cell Signaling Technology (USA). Rat anti-LAMP1 (1D4B) was obtained from BD Biosciences (USA). Rabbit anti-actin was obtained from Bethyl Laboratories (USA). Rabbit anti-GFP and rabbit anti-myc antibodies were made in our laboratory by immunizing rabbits with purified GFP protein and Myc epitope tag peptide (EQKLISEEDL), respectively.

### DNA Constructs

Following plasmids were previously described: pMSCVneo-UNC93B1-flag-TEV-HA (referred to as UNC93B1-HA), pMSCVpuro-TLR7-myc, pMSCVpuro-TLR9-myc, pMSCVpuro-UNC93B1-GFP (10, 19). Mutation of UNC93B1 (N251Q, N272Q, N449Q, N251Q/N272Q) was performed by site-directed mutagenesis PCR using pMSCVneo-UNC93B1-flag-TEV-HA as the template. Primers carrying the point mutation of AAC (Asn) to CAG (Gln) were used in the mutagenesis PCR.

### Preparation of Bone Marrow-Derived Dendritic Cells

Bone marrow-derived dendritic cells (BMDCs) were prepared as previously described (40). Briefly, femur and tibia from mice were flushed using a syringe and DMEM media. Flushed bone marrow cells were resuspended in DMEM media and red blood cells were eliminated by ACK lysis buffer treatment for 4 min, RT. Cells were then plated in 12-well plate at 1.5-2 x 10^6^ cells/ml density or in 24-well plate at 1 x 10^6^ cells/ml density. For BMDC differentiation, DMEM media supplemented with 10% FBS (Gibco, USA), 1% J588L/GM-CSF cell culture supernatant, 1% penicillin/streptomycin (Welgene, Korea), 55 μM β-mercaptoethanol (Sigma, USA) was used as culture media. A half of the culture media was changed with fresh media every 2 days until experiment on day 5 or 6.

### Retroviral Transduction

HEK293T cells were transfected with retroviral expression constructs of interest along with plasmids encoding gag/pol and VSV-G for retrovirus production. 36, 52 and 68 h after transfection, media containing virus particles was collected and spun at 3000 rpm for 5 min. Virus-containing media were added to iBMDMs or BMDCs at culture day 1 with 8 μg/mL polybrene (Sigma, USA). Cells were spun at 2200 rpm for 90 min and given fresh media after 16 h incubation. Cells stably expressing desired proteins were selected by culturing them in the presence of 500 μg/ml G418 (Sigma, USA) and/or 5 μg/ml puromycin (Sigma, USA) or sorted based on the GFP expression using BD FACSAria II (BD Biosciences, USA).

### Protein Deglycosylation

HEK293T cells expressing wild type or mutant UNC93B1-HA were harvested and lysed with lysis buffer containing 50 mM Tris-HCl (pH 7.5), 150 mM NaCl, 5 mM EDTA, 1% Triton X-100, and protease inhibitors for 30 min at 4°C. Cell lysates were spun down to remove cell debris and protein quantification was performed with a BCA protein assay kit (Thermo Fisher Scientific, USA) following manufacturer’s instruction. Protein deglycosylation with PNGase F (New England BioLabs, USA) was done following the manufacturer’s instruction. Briefly, cell lysates were denatured by addition of 10 x Glycoprotein Denaturing Buffer and incubation at 37°C for 30 min. The cell lysates were then treated with PNGase F and incubated at 37 °C for 30 min.

### NF-κB Reporter Assay

NF-κB reporter assay was performed as previously described (40). Briefly, HEK293T cells were co-transfected with expression plasmids for UNC93B1-HA and TLR-myc together with pcDNA3.1-NF-κB-luciferase in 6-well plates. Twenty four hours after transfection, cells were harvested and seeded in 96-well plates. Forty eight hours after transfection, cells were stimulated with TLR agonists for indicated time. Cells were then lysed with lysis buffer containing 50 mM Tris-HCl (pH 7.5), 150 mM NaCl, 5 mM EDTA, and 0.1% Triton X-100. D-luciferin solution (Sigma-Aldrich, USA) was added as a luciferase substrate and luciferase activity was analyzed with a luminometer (Labsystems, USA).

### IL-8 ELISA Assay

HEK293T cells were co-transfected with expression plasmids for UNC93B1-HA and TLR9-myc. Sixteen hours after transfection, cells were simulated with indicated concentration of CpG DNA for 24h. IL-8 in the culture supernatant was measured using an ELISA kit (BD Biosciences, USA) following manufacturer’s instruction.

### Intracellular Cytokine Assay

UNC93B1 KO BMDCs reconstituted with wild type or mutant UNC93B1-GFP were treated with 10 μg/mL brefeldin A and indicated concentration of CpG DNA for 4 h. Cells were fixed with 3.7% formaldehyde and permeabilized with 0.5% saponin-containing FACS buffer (2% BSA, 0.1% sodium azide in PBS). Then, cells were stained with Alexa Flour 647-conjugated anti-TNF antibody (BD Biosciences, USA), phycoerythrin (PE)-conjugated anti-IL-6 antibody (BD Biosciences, USA), and BD Horizon V450-conjugated anti-IL-12 antibody (BD Biosciences, USA) for 30 min, washed three times with FACS buffer, and analyzed with BD Fortessa (BD Biosciences, USA). The flow cytometry data were analyzed with FlowJo software (TreeStar, USA)

### Confocal Microscopy

UNC93B1 KO BMDCs were plated in 8-well Lab-Tek II chambered coverglasses (Nunc, USA) and transduced with retroviruses for fluorescent protein expression. Live cells were imaged with a spinning disk confocal microscope using Metamorph software (Molecular Devices, USA). UNC93B1 KO iBMDMs stably expressing TLR9-myc and wild type or mutant UNC93B1-GFP were plated on poly-lysine coated coverglasses in 24-well plates, fixed with 3.7% formaldehyde, and permeabilized with 0.3% Triton X-100 in staining buffer (2% BSA in PBS). Cells were stained with mouse anti-myc antibody (clone 9B11, Cell Signaling Technology, USA) and subsequently with Alexa Fluor 647-conjugated goat anti-mouse IgG (Invitrogen, USA). The coverglasses were mounted on microscope slides and cells were imaged with a confocal microscope, LSM780 (Zeiss, Germany) or A1 (Nikon, Japan).

### Phagosome Isolation

BMDMs were treated with magnetic beads (1μm diameter, Thermo Fisher Scientific, USA) and allowed to phagocytose the beads at 37 °C for 4 h. Cells were then washed twice with PBS, harvested in homogenization buffer (250 μM sucrose, 3 mM imidazole, pH 7.4), and homogenized by passage through 21G syringe for 25 strokes. Phagosomes containing the magnetic beads were isolated using IMag Cell Separation Magnet (BD Biosciences, USA) and washed twice with homogenization buffer. Phagosomes were lysed with lysis buffer containing 50 mM Tris-HCl (pH 7.5), 150 mM NaCl, 5 mM EDTA, 1% Triton X-100, and protease inhibitors at 4 °C for 15 min. Lysates were spun down to clear debris, and protein quantification was performed with a BCA protein assay kit (Thermo Fisher Scientific, USA) following manufacturer’s instruction.

### Immunoprecipitation and Immunoblotting

Cells were lysed with lysis buffer containing 50 mM Tris-HCl (pH 7.5), 150 mM NaCl, 5 mM EDTA, 1% digitonin, and protease inhibitors for 1 h at 4 °C. Cell lysates were spun down to remove cell debris and protein quantification was performed with a BCA protein assay kit (Thermo Fisher Scientific, USA). Cell lysates or phagosomal lysates were incubated with an anti-myc antibody (9B11) at 4 °C, overnight and subsequently with Protein A-conjugated agarose beads (Repligen, USA) at 4 °C for 4 h. The agarose beads were washed with lysis buffer three times and the co-immunoprecipitated proteins were subjected to SDS-PAGE and immunoblotting with rat anti-HA (3F10), rabbit anti-GFP, or rabbit anti-MyD88 (D80F5) antibody.

## Data Availability Statement

The raw data supporting the conclusions of this article will be made available by the authors, without undue reservation.

## Ethics Statement

The animal study was reviewed and approved by the Institutional Animal Care and Use Committee of KAIST and the Institutional Animal Care and Use Committee of POSTECH.

## Author Contributions

H-SS, J-WH, and Y-MK conceived and designed the experiments. H-SS, SP, J-WH, Y-RL, D-JJ, CY, SK, and HK performed the experimental works. H-SS, SP, J-WH, Y-RL, D-JJ, CY, and Y-MK analyzed and interpreted the results. H-SS and Y-MK drafted the manuscript. Y-MK is the guarantor of integrity of the entire study and responsible for the article. All authors contributed to the article and approved the submitted version.

## Funding

This study is supported by grants from the National Research Foundation of Korea (2017M3A9F3047085, 2020R1A2C2011307) and Korea Advanced Institute of Science and Technology.

## Conflict of Interest

The authors declare that the research was conducted in the absence of any commercial or financial relationships that could be construed as a potential conflict of interest.

## Publisher’s Note

All claims expressed in this article are solely those of the authors and do not necessarily represent those of their affiliated organizations, or those of the publisher, the editors and the reviewers. Any product that may be evaluated in this article, or claim that may be made by its manufacturer, is not guaranteed or endorsed by the publisher.

## Supplementary Material

Supplementary Figure 1 Glycosylation at N272 of UNC93B1 is not required for TLR7 signaling. **(A, B)** GFP or UNC93B1-GFP (wild type or mutant) was expressed in UNC93B1 KO BMDCs. After stimulation of cells with indicated concentration of R848 for 4 h, cytokine production was analyzed by intracellular cytokine staining followed by flow cytometry. Representative FACS plots for TNF-α, IL-6, and IL-12 in GFP^+^ BMDCs stimulated with 50 nM R848 are shown **(A)**. The proportion of TNF-α ^+^, IL-6^+^, and IL-12^+^ cells among GFP^+^ BMDCs are shown **(B)**. **(C)** UNC93B1 KO iBMDMs expressing wild type or mutant UNC93B1-GFP were stimulated with indicated TLR agonists for 4 h and TNF-α production was analyzed by intracellular cytokine staining followed by flow cytometry. The proportion of TNF-α ^+^ cells are shown. Data represent means ± SD (n = 3). *p < 0.05, **p < 0.01 (Student’s *t*-test)

Supplementary Figure 2 Structure comparison of TLRs in complex with UNC93B1. **(A)** Superposition of the TLR3-UNC93B1 complex (PBB 7C76, green for TLR3 and pink for UNC93B1) and the monomeric TLR7-UNC93B1 complex (PDB 7CYN, blue for TLR7) using UNC93B1 as reference. Due to the absence of available structure for TLR9-UNC93B1 complex, the LRRCT domain (C765-Q805) of TLR9 ectodomain (PDB 3WPF, yellow for TLR9) is aligned to TLR7 LRRCT domain (C787-L828), and TLR9 transmembrane region is modeled using TLR3 transmembrane region as a template. Glycans on UNC93B1 (N251 and N272) and TLRs are indicated as spheres and colored with light brown, red, and orange, respectively. Glycans on TLR7 are adopted from high-resolution structure of TLR7 ectodomain (PDB 6LVZ). **(B)** The TLR3-UNC93B1 complex. **(C)** The monomeric TLR7-UNC93B1 complex. **(D)** The modeled TLR9-UNC93B1 complex. The solid lines and dotted lines represent the ring plane of each TLR ectodomain and the plane perpendicular to the membrane, respectively. The degrees by which the two planes are separated are depicted.
