# Supplementary figures and images for: N-glycosylation of UNC93B1 at a Specific Asparagine Residue Is Required for TLR9 Signaling
Source: Front Immunol. 2022 Jul 7;13:875083. doi: 10.3389/fimmu.2022.875083 (PMC9301129; doi:10.3389/fimmu.2022.875083)

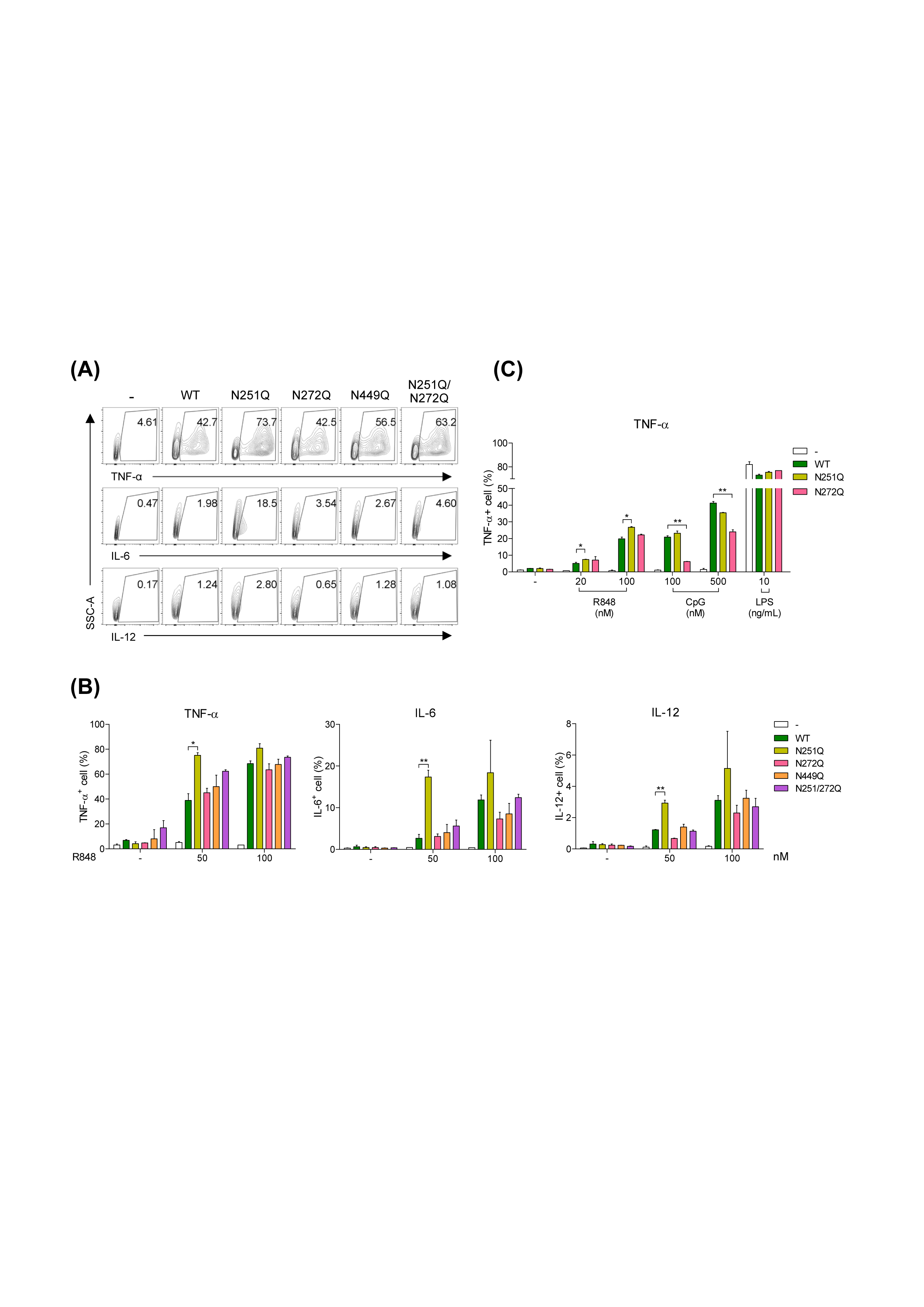

Supplement: Supplementary Figure 1 — Glycosylation at N272 of UNC93B1 is not required for TLR7 signaling. (A, B) GFP or UNC93B1-GFP (wild type or mutant) was expressed in UNC93B1 KO BMDCs. After stimulation of cells with indicated concentration of R848 for 4 h, cytokine production was analyzed by intracellular cytokine staining followed by flow cytometry. Representative FACS plots for TNF-α, IL-6, and IL-12 in GFP+ BMDCs stimulated with 50 nM R848 are shown (A). The proportion of TNF-α +, IL-6+, and IL-12+ cells among GFP+ BMDCs are shown (B). (C) UNC93B1 KO iBMDMs expressing wild type or mutant UNC93B1-GFP were stimulated with indicated TLR agonists for 4 h and TNF-α production was analyzed by intracellular cytokine staining followed by flow cytometry. The proportion of TNF-α + cells are shown. Data represent means ± SD (n = 3). *p < 0.05, **p < 0.01 (Student’s t-test) [file Image_1.tif]

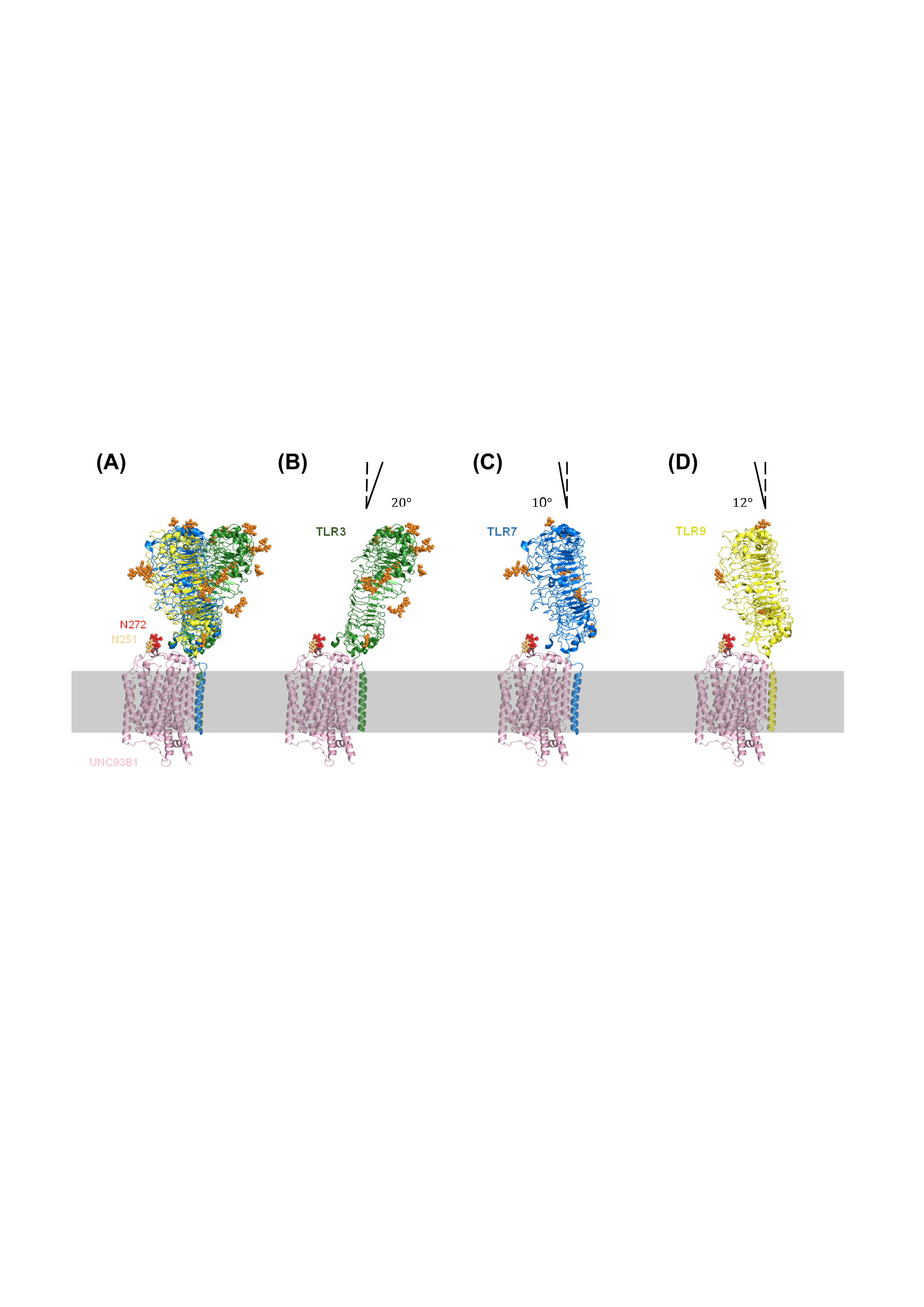

Supplement: Supplementary Figure 2 — Structure comparison of TLRs in complex with UNC93B1. (A) Superposition of the TLR3-UNC93B1 complex (PBB 7C76, green for TLR3 and pink for UNC93B1) and the monomeric TLR7-UNC93B1 complex (PDB 7CYN, blue for TLR7) using UNC93B1 as reference. Due to the absence of available structure for TLR9-UNC93B1 complex, the LRRCT domain (C765-Q805) of TLR9 ectodomain (PDB 3WPF, yellow for TLR9) is aligned to TLR7 LRRCT domain (C787-L828), and TLR9 transmembrane region is modeled using TLR3 transmembrane region as a template. Glycans on UNC93B1 (N251 and N272) and TLRs are indicated as spheres and colored with light brown, red, and orange, respectively. Glycans on TLR7 are adopted from high-resolution structure of TLR7 ectodomain (PDB 6LVZ). (B) The TLR3-UNC93B1 complex. (C) The monomeric TLR7-UNC93B1 complex. (D) The modeled TLR9-UNC93B1 complex. The solid lines and dotted lines represent the ring plane of each TLR ectodomain and the plane perpendicular to the membrane, respectively. The degrees by which the two planes are separated are depicted. [file Image_2.tif]
